# Synthesis, *in-Vitro* and *in Silico* Studies of Azo-Based Calix[4]arenes as Antibacterial Agent and Neuraminidase Inhibitor: A New Look Into an Old Scaffold

**DOI:** 10.3389/fchem.2018.00210

**Published:** 2018-06-12

**Authors:** Yousaf Ali, Noraslinda Muhamad Bunnori, Deny Susanti, Alhassan Muhammad Alhassan, Shafida Abd Hamid

**Affiliations:** ^1^Department of Chemistry, Sarhad University of Science and Information Technology, Peshawar, Pakistan; ^2^Kulliyyah of Science, International Islamic University Malaysia, Kuantan, Malaysia; ^3^Kulliyah of Pharmacy, International Islamic University Malaysia, Kuantan, Malaysia

**Keywords:** calix[4]arenes, azo calix[4]arenes, antibacterial activity, docking, neuraminidase inhibition

## Abstract

Calixarene derivatives are reported as potential therapeutic agents. Azo derivatives of calixarenes have not been given much consideration to explore their biomedical applications. In the present study, some azo-based derivatives of calix[4]arene were synthesized and characterized and their antibacterial and antiviral potentials were studied. The mono azo products of sulphanilamide, sulfaguanidine and 2-methyl-4-aminobenzoic acid showed good activity against bacterial strains with minimum inhibition concentration values ranging from 0.97 to 62.5 μg/mL. For mono azo products, the diazotized salt was applied as a limiting reagent. The use of calix[4]arene and sodium acetate trihydrate in 1:3 (molar ratio) helped in partial substitution. Molecular docking was performed to see the interaction of the designed compounds with two bacterial and one viral (neuraminidase) receptor. Some of the derivatives showed good interaction with the active site of bacterial and neuraminidase enzymes through hydrogen, hydrophobic and pi-pi interactions, and could inhibit the activity of the selected enzymes.

## Introduction

Calixarenes, known as the third generation of supramolecules, are cyclic oligomers that are synthesized from phenol and formaldehyde (Shinkai, 1993). Calixarene derivatives have been described as metal extractants, ions transporters, electrode ionophores optical sensors (Vicens and Böhmer, 2012; Deska et al., 2015), while various others have been suggested as potential drug candidates (Yousaf et al., 2015). They showed antimicrobial, antidiabetic, antitubercular, anticancer and anti-HIV activities (Nimse and Kim, 2013; Tauran et al., 2015). The cup-shaped structure of calixarene also provides suitable architecture to be used as drug delivery vehicle (Mo et al., 2016).

Calixarene derivatives containing azo moiety (-N = N-) are synthesized by insertion of nitrogen at the *para* position(s) of de-butylated calixarene or by incorporating phenyl azo moieties at lower rim via the phenolic hydroxy group (Deligöz and Ercan, 2002; Sliwa and Deska, 2011). The four *para* positions of calix[4]arene are equally available for nitrogen insertion to give the tetrakis azo product. Due to the symmetry of the calix[4]arene, it is quite a challenge to introduce functional groups in a selective fashion among the four *p*ara positions (Vicens et al., 2007). Selective diazotization at the upper rim is mostly reported after lower rim modification such as alkylation, acylation etc. (Karakuş and Deligöz, 2015). Steric hindrance of the coupling agent and incompletion of reaction have also been reported as the reasons for partial substitution (Ramanjaneyulu et al., 2010). To obtain mono-(*p-*substituted phenyl)azo calix[4]arene in quantitative yield, Jin et al. (2002) used isoamyl nitrite in EtONa/EtOH for the diazotization of aniline and the reaction was carried out in the presence of carbon dioxide gas in THF.

**Figure 1:**
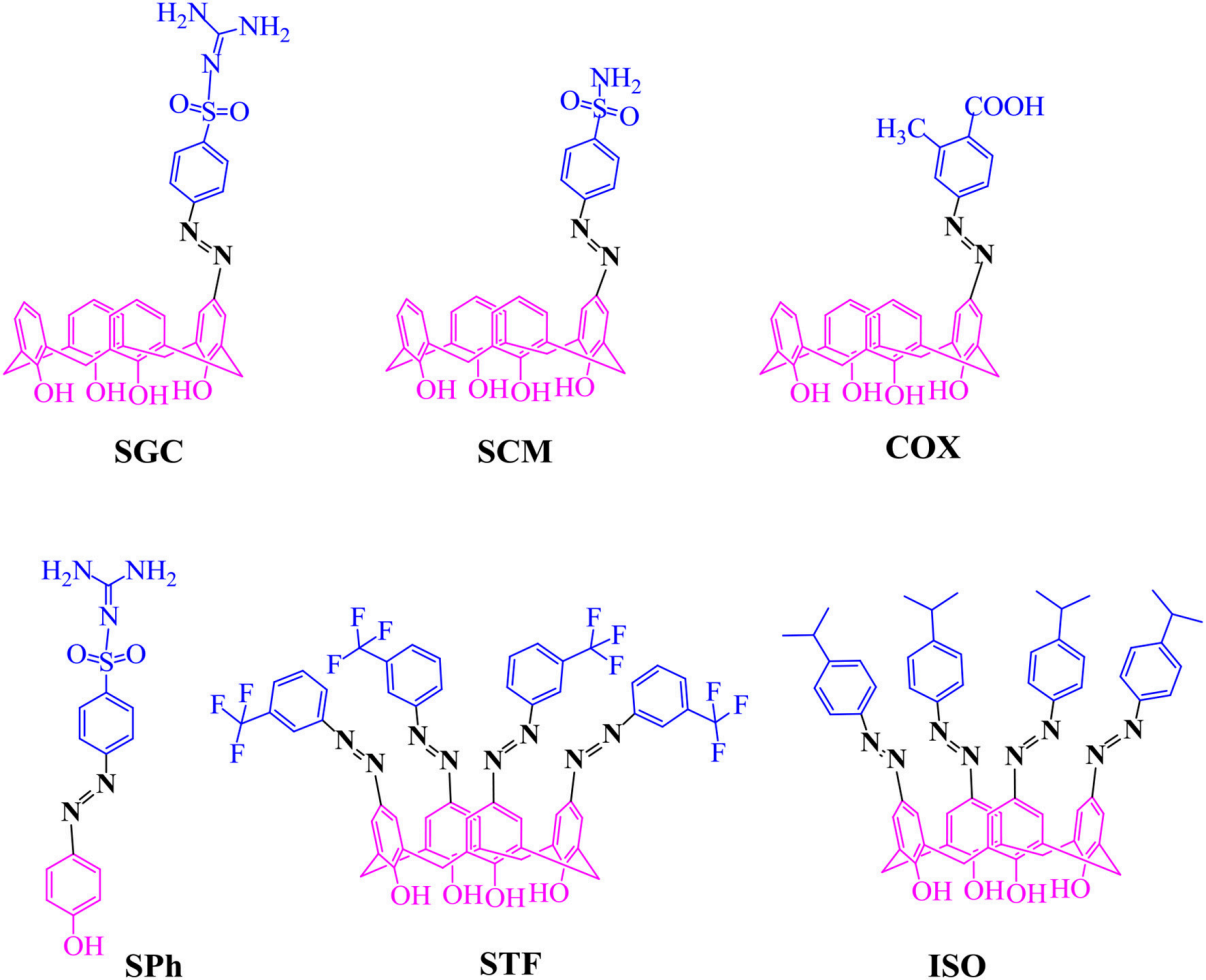
Chemical structures of synthesized azo compounds.

Azo calixarenes are mainly reported for detection of different ionophores and extraction of transition metal cations (Elçin et al., 2016). Some azo calixarenes also showed coloring properties (Tang et al., 2015). However, the drug-like potential of azo calixarenes has not been given much consideration. A*zo compounds of pyrimidine (*Gulcan et al., 2014*;* Shaikh and Meshram, 2015*) and other therapeutically recognized classes of organic compounds, such as enamines, pyrazole, thiazole, and triazole have shown excellent antimicrobial activities (*Rizk et al., 2015*;* Sahoo et al., 2015*)*. It has been reported that the introduction of azo group has improved more than 60% of the antibacterial activities of certain molecules (Mkpenie et al., 2008). It was assumed that azo calix[4]arenes will also show better antimicrobial properties. Some upper rim-modified azo calix[4]arenes were synthesized (Figure 1) and their activity against selected strains of bacteria and fungi was evaluated. An optimized protocol was applied to obtain mono azo calix[4]arene in reasonable yield. Molecular docking study was performed to see the binding interaction and inhibition potential of the selected compounds toward the microbial enzymes. In addition, docking against neuraminidase receptor was also included as a theoretical model of antiviral study.

## Materials and methods

### Synthesis

All the chemicals and reagents were supplied by Acros Organics, Merck, Sigma-Aldrich, or QReC and used without further purification. Melting point analysis was carried out by open capillaries using Electrothermal melting point apparatus (UK). ^1^H-NMR and ^13^C-NMR spectra of the compounds were recorded on BRUKER AVANCE III ULTRASHIELD PLUS spectrometer at 500 and 125 MHz, respectively (Supporting Information). Deuterated solvents of different choices based on the solubility of compounds were used. Chemical shifts are given in δ scale (ppm) and tetramethylsilane (TMS) was used as internal reference. Nominal mass spectra of selected compounds were analyzed on LcQ Finnigan MAT mass spectrometer with atmospheric pressure chemical ionization (APCI) as a probe.

#### 25,26,27,28-tetrahydroxyclaix[4]arene

1 g of *p-tert-*butylcalix[4]arene (1.54 mmol) and 0.8 g of phenol (8.49 mmol) were stirred in dry toluene (10.5 mL) under nitrogen for 45 min at 90°C. The suspension was allowed to cool and AlCl_3_ (1.1 g, 8.23 mmol) was added at 5–10°C while stirring. After 12 h stirring at room temperature, 2 M HCl (6.5 mL) was added to the mixture and stirring was continued for further 30 min. The organic layer was separated and the excess toluene was evaporated by rotary evaporator. Chloroform (4.5 mL) was added to the flask and refluxed for 30 min to give a clear solution which was allowed to cool. The product precipitated after addition of MeOH (6.5 mL). Recrystallization from CHCl_3_/MeOH (6:2) gave white powder product. Yield (45%), mp 312–313°C; ^1^H NMR (500 MHz, CDCl_3_) δ (ppm): 3.50 (4H, bs, Ar-CH_2_-Ar), 4.32 (4H, bs, Ar-CH_2_-Ar), 6.71 (4H, t, Ar-H, *J* = 8.0 Hz), 7.04 (8H, d, Ar-H, *J* = 12.0 Hz), 10.2 (s, 4H, Ar-OH); ^13^C-NMR (125 MHz, CDCl_3_) δ: 148.8, 129.0, 128.2, 122.3, 31.7.

#### Synthesis of N-(diaminomethylidene)-4-[(E)-(4-hydroxyphenyl)diazenyl] benzenesulfonamide (SPh)

A solution of sodium nitrite (0.17 g, 7.3 mmol) in water (2 mL) was slowly added to an acidified solution of sulfaguanidine (0.64 g, 3 mmol) in water/acetone mixture (12 mL, 2:1) at 0°C. The resulting diazonium salt solution was gently added to an ice-cold solution of phenol (0.29 g, 3 mmol), NaOH (0.34 g, 8.5 mmol) and Na_2_CO_3_ (0.9 g, 8.5 mmol) in 10 mL water and the mixture was stirred for 2 h. Then 50 mL water was added and the mixture was allowed to stand for 2 h. The yellow precipitate was filtered, recrystallized from EtOAc and purified by column chromatography (CHCl_3_/MeOH, 4:1). Yield (67%), mp 256–258°C; IR (KBr) υ: 1541.5 cm^–1^ (-N = N), ^1^H-NMR (500 MHz, DMSO-d_6_) δ (ppm): 6.77 (4H, bs, C-*NH*_2_), 6.9 (2H, d, Ar-H, *J* = 8.0 Hz),7.8 (2H, d, Ar-H, *J* = 10.0 Hz), 7.9 (4H, d, Ar-H, *J* = 8 Hz), ^13^C-NMR (125 MHz, DMSO-*d*_6_) δ: 161.6, 158.1, 153.2, 145.1, 126.8, 125.2, 116.0.

#### 25,26,27,28-tetrahydroxy-5-(sulfaguanidine)azocalix[4]arene (SGC)

A solution of diazonium salt, prepared from sulfaguanidine (0.2 g, 1 mmol), sodium nitrite (0.069 g, 1 mmol) and conc. HCl (2 mL) in 10 mL water/acetone (1:2) mixture, was added to an ice-cold solution of calix[4]arene (0.85 g, 2 mmol) and sodium acetate trihydrate (0.68 g, 5 mmol) in H_2_O/DMF (20 mL, 5:8, v/v) giving yellowish orange suspension. After standing for 2 h at room temperature, the suspension was acidified with aqueous HCl (50 mL, 0.25%). The dried solid mass was dissolved in dichloromethane and purified by column chromatography (Hex/EtOAc, 2:3). Yield (55%), mp 263-65°C; IR (KBr) υ: 1,541 cm^–1^ (-N = N-), ^1^H-NMR (500 MHz, CDCl_3_) δ (ppm): 3.55 (4H, bs, Ar-CH_2_-Ar), 4.28 (4H, bs, Ar-CH_2_-Ar), 6.5 (4H, bs, C-*NH*_2_), 6.71 (3H, t, Ar-H, *J* = 7.5 Hz), 7.04 (6H, d, Ar-H, *J* = 8 Hz), 7.6 (2H, s, Ar-H(calix)), 7.8 (4H, d, Ar-H, *J* = 8.5 Hz), 7.9 (4H, d, Ar-H, *J* = 8.5 Hz); ^13^C-NMR (125 MHz, DMSO-*d*_6_) δ: 159.8, 158.0, 153.5, 149.3, 144.9, 144.5, 130.6, 128.5, 126.6, 124.0, 122.0, 121.1, 30.6, 30.5; ESI-MS *m/z* calcd. for [C_35_H_31_N_5_O_6_S]^+^ 649.20; found 648.30 [M+H]^+^.

#### 25,26,27,28-tetrahydroxy-5-(4-sulphonylaminophenyl)azocalix[4]arene (SCM)

Compound **SCM** was prepared as described above for compound **SGC**. The diazonium salt of 4-aminobenzenesulfonamide (0.172 g, 1 mmol), was added to an ice-cold solution of calix[4]arene (0.85 g, 2 mmol) and sodium acetate trihydrate (0.68 g, 5 mmol) in H_2_O/DMF (20 mL, 5:8, v/v). After standing for 2 hr at room temperature, the suspension (red color) was acidified with aqueous HCl (50 mL, 0.25%) to give the orange solid product, which was filtered, washed with distilled water and dried over MgSO_4_. The product was purified by column chromatography (CHCl_3_/MeOH, 7:1). Yield (67%), >200 mp°C; IR (KBr) υ: 1,590 cm^–1^ (-N = N-), ^1^H-NMR (500 MHz, CDCl_3_) δ (ppm): 3.56 (bs, Ar-CH_2_-Ar);), 4.29 (4H, bs, Ar-CH_2_-Ar), 4.89 (2H, s, OH) 6.9 (4H, bs, C-*NH*_2_), 6.74 (3H, m, Ar-H), 7.1 (6H, m, Ar-H), 7.73 (2H, s, Ar-H(calix)), 7.9 (4H, d, Ar-H, *J* = 8.4 Hz), 8.0 (4H, d, Ar-H, *J* = 10.6 Hz), 10.19 (4H, s, OH); ^13^C-NMR (125 MHz, DMSO-*d*_6_) δ: 150.2, 129.4, 129.2, 129.0, 128.6, 127.2, 127.5, 122.5, 121.4, 121.3, 31.2,31.1; ESI-MS *m/z* calcd. for [C_34_H_29_N_3_O_6_S]^+^ 607.18; found 606.2 [M-H]^+^.

#### 25,26,27,28-tetrahydroxy-5-((4-corboxy-3-methyl)phenyl)azocalix[4]arene (COX)

Compound **COX** was prepared as described above for compound **SGC**. The diazonium salt, of 4-amino-2-methylbenzoic acid (0.15 g, 1 mmol) was added to an ice-cold solution of calix[4]arene (0.85 g, 2 mmol) and sodium acetate trihydrate (0.68 g, 5 mmol) in H_2_O/DMF (20 mL, 5:8, v/v). The product was purified by preparative chromatography (Hex/EtOAc, 2:3). Yield (48%), mp 260-62°C; IR (KBr) υ: 1591 cm^–1^ (-N = N-), ^1^H-NMR (500 MHz, MeOD) δ (ppm): 3.30 (8H, s, Ar-CH_2_-Ar), 6.58 (3H, t, Ar-H, *J* = 7.5 Hz), 6.96 (1H, d, Ar-H, *J* = 7.5 Hz), 7.03 (6H, m, Ar-H, *J* = 7.5 Hz), 7.54 (1H, d, Ar-H, *J* = 8.1 Hz), 7.58 (1H, s, Ar-H), 7.65 (2H, s, Ar-H): ^13^C-NMR (125 MHz, MeOD) δ*:* 154.5, 153.5, 152.4, 138.6, 129.4, 125.3, 121.3, 120.09, 43.1,32.8, 21.2; ESI-MS *m/z* calcd. for [C_36_H_30_N2O_6_]^+^ 586.21; found 585.2 [M-H]^+^.

#### 25,26,27,28-tetrahydroxy-5,11,17,23-tetrakis(3-trifluoromethyl)phenyl) azocalix[4]arene (STF)

A solution of diazonium chloride, prepared from 3(trifluoro)methylaniline (1.28 g, 8 mmol), sodium nitrite (1.1 g, 16 mmol) and conc. HCl (2 mL) in water (5 mL), was added dropwise to a cold (5°C) solution of calix[4]arene (1.7 g, 4 mmol) and sodium acetate trihydrate (6.8 g, 50 mmol) in H_2_O/DMF (25 mL, 5:8 v/v) to give an orange suspension. The suspension was acidified with aqueous HCl (80 mL, 0.25%) and the mixture was then warmed to 60°C for 30 min. Then 50 mL EtOAc was added to the mixture at room temperature. The crude product gave a pale brown solid, which was filtered and washed with EtOAc and purified by column chromatography (Hex/EtOAc, 1:3). Yield (75%), mp >280°C; ^1^H-NMR (500 MHz, CDCl_3_) δ (ppm): 3.87 (4H, bs, Ar-CH_2_-Ar), 4.41 (4H,bs, 4H, Ar-CH_2_-Ar), 7.59 (6H, t, Ar-H, *J* = 7.7 Hz), 7.67 (1H, d, Ar-H(calix), *J* = 7.65 Hz), 7.85 (4H, s, Ar-H), 8.00 (2H, d, Ar-H, *J* = 8.0 Hz), 8.10 (4H, s, Ar-H), 10.28 (4H, s, OH); ^13^C-NMR (125 MHz, DMSO-d_6_) δ: 159.1, 152.2, 144.5, 130.5, 130.2, 129.8, 126.06, 122.7, 117.6, 31.52; ESI-MS *m/z* calcd. for [C_56_H_36_F_12_N_8_O_4_]^+^ 1112.27; found 1113.1 [M+H]^+^.

#### 25,26,27,28-tetrahydroxy-5,11,17,23-tetra(4-isopropylphenyl)azocalix[4]arene (ISO)

Compound **ISO** was prepared as described for compound **CTF** by adding diazonium salt of isopropylaniline (0.48 g, 3.5 mmol) to an ice cold (5°C) solution of calix[4]arene (0.212 g, 0.5 mmol) and sodium acetate trihydrate (2.38 g, 17.5 mmol) in H_2_O/DMF (25 mL, 5:8, v/v) to give gray suspension. The crude product gave a pale brown solid which was purified by coloumn chromatography (Hex/EtOAc, 2:1). Yield (72%), mp >280°C; IR (KBr) υ: 1,469 cm^–1^ (-N = N-), ^1^H-NMR (500 MHz, CDCl_3_) δ (ppm): 1.25 (24H, d, C-(CH_3_)_2_, *J* = 7.0 Hz), 2.85 (4H, q, -CH(CH_3_)_2_, 3.84 (bs, Ar-CH_2_-Ar);), 4.37 (bs, 4H, Ar-CH_2_-Ar), 7.30 (4H, d, Ar-H, *J* = 8.35 Hz), 7.75 (4H, d, Ar-H, *J* = 8.4 Hz), 7.78 (8H, s, Ar-H(calix), 10.25(4H, s, OH), ^13^C-NMR (125 MHz, CDCl_3_) δ*:* 151.85, 151.1, 147.8,128.2, 127.02, 124.2, 122.6, 34.09, 31.8, 23.8. ESI-MS *m/z* calcd. for [C_64_H_64_N_8_O_4_]^+^ 1008.51; found 1009.3 [M+H]^+^.

### *In vitro* antibacterial assay

The MIC values of the synthesized azo calix[4]arenes were determined using micro broth dilution method (Balouiri et al., 2016). Briefly, morphologically identical 3–4 colonies of cultured bacteria were taken from agar plates and suspended in a 4–5 mL of Mueller–Hinton broth (MHB). The suspension was incubated at 37°C for 2–6 h and then diluted to adjust the size of inoculum to the 0.5 MacFarland standard turbidity, 5 × 10^5^-5 × 10^6^ CFU/mL (Colony Forming Unit). The stock solutions of the compounds were serially diluted to obtain 125, 62.5, 31.2, 15.6, 7.8, 3.9, 1.9, and 0.97 μg mL^−1^ concentrations. An equal volume of bacterial inoculum was added to each well containing 0.05 mL of compound's serial dilutions. After incubation for 20–24 h at 37°C, MIC was determined with micro plate reader as the lowest concentration of tested compound with absorbance that was comparable with the control/reference wells. Wells without inoculum or broth with only the drug were used as negative control. Gentamycin/chloramphenicol and bacteria-free solvent were used as a positive and negative control, respectively. Nystatin was used as reference drug for antifungal study. The MBC was determined by culturing the contents of well exhibited no growth onto agar plates. The plates were incubated at 35°C for 24 h. The MBC was considered as the lowest concentration of compound required to kill (99.9%) of the tested microbes. The experiments were performed in triplicate and repeated three times with similar results.

### Docking procedure

Docking was performed by Autodock4.2. The crystal structures of receptors (PDB IDs: 4CJN, 1CEF and 3TI6) were retrieved from the Protein Data Bank (http://www.rcsb.org/pdb). The heteroatoms and water molecules were removed from protein. The PDB file of the receptor was added hydrogen bonds and the Gasteiger charge. Autogrid was used to obtain pre-calculated grid maps and the default optimization parameters were used (a maximum number of 250,000 energy evaluations and a maximum number of generations of 27,000), except the number of GA runs was increased to 30–50. The grid box used for specifying the search space was centered on the active site of the receptor with a default grid point spacing of 0.375 Å. To get more stable configuration, the energy of ligands was minimized to get more stable configuration. The docking result is presented in terms of binding energy (B.E) values given in kcal/mol and inhibition constant (Ki) in micro molar (μM). The pose/conformation with the lowest B.E and the appropriate cluster was considered for further analysis and visualization. The molecular visualization of the docked complexes was performed using the Accelrys Discovery Studio 2016.

## Results and discussion

### Chemistry

Tetrahydrocalix[4]arene was synthesized following the general procedure (Gutsche et al., 1985; Gutsche and Iqbal, 1990). Usually, molecules with small size and low molecular weight are preferred in drug designing process (Schneider, 2000–2013). Mono azo products have low molecular weight and small size, and hence considered more close to the Lipinski Rule of 5. These factors are usually considered in drug designing process. The desired mono azo products were synthesized in reasonable yield according to the reported procedure (Morita et al., 1992; Elçin et al., 2013) with slight modification. The diazotized salt was applied as a limiting reagent and added to the basic solution of calix[4]arene at once instead of slow addition. Calix[4]arene and sodium acetate trihydrate were used in 1:3 (molar ratio) rather than 1:15. The latter is commonly practized by other researchers for the synthesis of the tetrakis azo product of calix[4]arene (Elçin et al., 2013). The reactions were stopped immediately (2–3 min) after addition of diazonium salt in all of the compounds, except in the case where tetra- product was desired. The combined effect of these three conditions resulted in mono azo calix[4]arene as the main product. The only disadvantage of selective diazotization by this method was an excess of unreacted calix[4]arene left after completion of the reaction, as underlined by other researchers as well (Chawla et al., 2006). However, the starting material was easily recovered by recrystallization or column chromatography. The use of weak catalyst like CsF was usually favored for mono alkylation of calix[4]arene at lower rim (Groenen et al., 1991). The same catalyst was used for upper rim modification replacing sodium acetate trihydrate that gave comparable result in producing a mono azo product of sulfanilamide.

Three mono azo calix[4]renes incorporating sulfaguanidine (**SGC**), sulphanilamide (**SCM**) and 2-methyl-4-amino benzoic acid (**COX**) moieties were synthesized (Figure 1) and the products were obtained in reasonable yield (40–65%). Taking calix[4]arene and sodium acetate trihydrate in 1:3 (molar ratio) might have a role in partial substitution giving mono azo product. This may be due to the formation of monoanion of the calix[4]arene when the weak base removed only a single proton from the calix[4]arene. The ^1^H-NMR spectrum of mono-substituted products showed a doublet at ~7.01 and a triplet at ~6.9 ppm for *ortho* and *para* hydrogens of phenolic rings of calix[4]arene, respectively. The singlet at ~7.5–7.9 ppm indicates that *para* position of one phenol ring of calix[4]arene was substituted by the azo moiety. The bridging methylene peaks appeared at ~3.52 and ~4.21 ppm reflect the existence of the product in the same (cone) conformation. The ^1^H-NMR spectra of **SGC** and **SCM** gave two additional peaks of doublet (7.8–8.2 ppm) for sulfanilamide and sulfaguanidine units. A peak around 2.5 ppm in **COX** represents the methyl group of the inserted azo moiety. The MS-ESI spectra of these compounds confirmed that the insertion of azo moiety has occurred at a single position (see Supporting Information).

**Figure 2:**
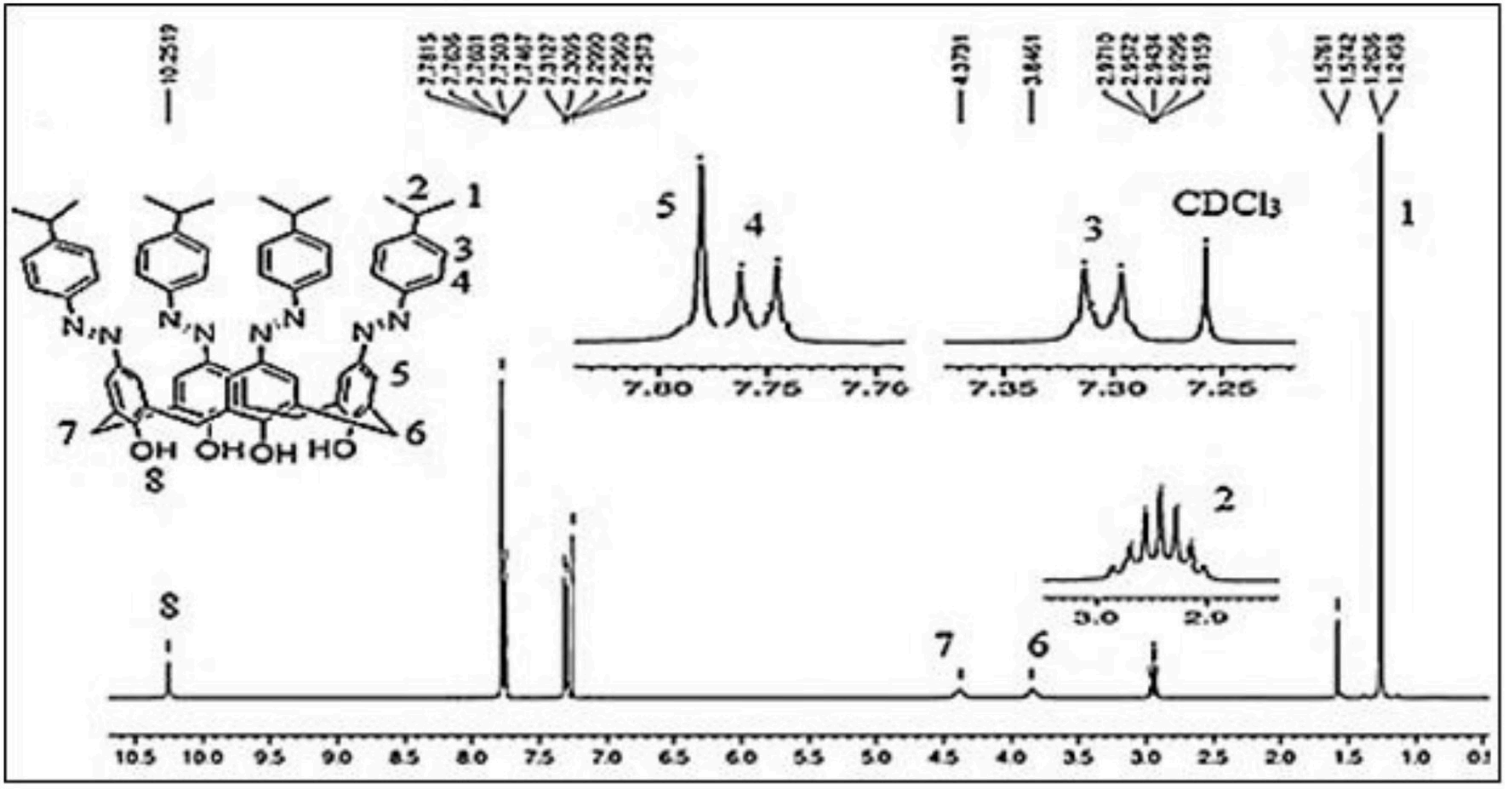
^1^H-NMR spectrum of compound **ISO** (5,11,17,23-tetrakis(4-isopropylphenyl) azo calix[4]arene).

The tetrakis azo products of isopropyl aniline (**ISO**) and 3-(trifluoro)methylaniline (**STF**) were produced by a reported method using sodium acetate trihydrate in MeOH-DMF (5:8) An alternative method using pyridine as a medium of the reaction was also adopted (Hamon et al., 2009), however, this approach was found less suitable in term of yield and purity of products. The absence of a doublet at ~7.01 and triplet at ~ 6.9 ppm in ^1^H-NMR spectra of these compounds indicate that all the *para* positions have been occupied by azo moieties. The ^1^H-NMR spectrum of **ISO** (Figure 2) showed a singlet at 7.8 ppm for the eight *ortho* hydrogens of calix[4]arene unit. In ^13^C-NMR, the two characteristic peaks of the product at 20 ppm and 33 ppm correspond to methyl groups and methine carbon. The IR spectrum of the compound showed two bands for CH_3_ and CH protons at 2,959 and 2,866 cm^−1^ respectively. In the case of **STF**, an additional singlet at 8.10 ppm appeared for the four *ortho* hydrogens of the azo moiety.

### *In vitro* study

The synthesized compounds were screened against five Gram-positive bacterial strains (Bacillus subtilis, *Staphylococcus aureus, Methicillin-resistant Staphylococcus aureus* (*MRSA), Staphylococcus epidermidis Enterococcus faecalis*)*, two* Gram-negative bacterial strains (Escherichia coli and *Pseudomonas aeruginosa*) and two fungal strains (*Candida albicans* and *Saccharomyces cerevisiae*). Table 1 reveals that most of the compounds showed activity only against Gram-positive strains. It may be due to the fact that Gram-negative bacteria and fungi have more complex structure than Gram-positive bacteria. The impenetrable cell membrane of Gram-negative bacteria is one of the reasons that many drugs are ineffective against these strains (Silhavy et al., 2010; Miller, 2016).

**Table 1:** MIC values of synthesized compounds against selected microbial strains.

**Microbes**	**SCM MIC (μg/mL)**	**SGC MIC (μg/mL)**	**COX MIC (μg/mL)**	**SPh MIC (μg/mL)**	**ISO MIC (μg/mL)**	**STF MIC (μg/mL)**	RD^**^
*S. aureus*	3.9	7.8	7.8	>125	>125	>125	G = 3.9
MRSA	0.97	15.6	7.8	>125	31.25	>125	Ch = 0.97 G > 62.5
*B. subtilis*	0.97	15.6	0.97	>125	125	>125	G < 0.5
*S. epidermidis*	1.9	7.8	1.9	>125	62.5	>125	G > 31.2
*E. faecalis*	7.8	31.25	7.8	>125	15.6	>125	G > 31.2
*E. coli*	>125	>125	125	>125	>125	>125	G < 31.2
*P. aeruginosa*	>125	15.6	62.5	>125	62.5	>125	G < 0.5
*C. albicans*	>125	62.5	>125	>125	>125	>125	Ny < 0.9
*S. cerevisiae*	>125	>125	>125	>125	>125	>125	Ny < 0.9

** *RD, reference drug; G, gentamycin; Ch, chloramphenicol; Ny, nystatin*.

Compound **SCM** showed better activity among the tested compounds. It has the lowest minimum inhibitory concentration (MIC) value against MRSA (0.97 μg/mL) and *B. subtilis* (0.97 μg/mL). The reference drug, chloramphenicol gave MIC value 0.97 μg/mL against MRSA while that of gentamycin (**G**) was found to be > 62.5 μg/mL. The compound also showed good inhibition against *S. aureus* (MIC 3.9 μg/mL), *S. epidermidis* (MIC 15.6 μg/mL) and *E. faecalis* (MIC 3.9 μg/mL). The activity of **SCM** may be attributed to the presence of sulfonamide unit. The antimicrobial activity of sulfonamides is due to its structural similarity with *p-*aminobenzoic acid (*p*ABA), which is produced by bacteria for the biosynthesis of folic acid, an essential growth factor. Sulfonamides exert antibacterial action by antagonizing *p*ABA utilization, thus prevent the synthesis of folic acid and stop bacterial growth (Achari et al., 1997). However, compound **SCM** has no effect on the selected Gram-negative and fungal strains. The complex nature of the cell wall may be the possible reason for their inactivity.

Sulfaguanidine-based azo calix[4]arene (**SGC**) showed good inhibition against *S. epidermidis, S. aureus* (MIC 7.8 μg/mL), MRSA and *B. subtilis* (MIC 15.6 μg/mL). In addition, it was also active against *P. aeruginosa* (Gram-negative) at MIC 15.6 μg/mL and *C. albicans* (fungus) at MIC 62.5 μg/mL. **SGC** was found to possess higher activity compared to its respective monomer, sulfaguanidine phenolic azo compound (**SPh**). The ratio between the MIC of monomer (**SPh**) and its corresponding azo calix[4]arene (**SGC**) ranged from 10 to 22 μg/mL. It indicates that the activity of **SGC** is due to the combined effect of guanidinium unit and macrocyclic scaffold. A similar result was published by Mourer et al. (2009), where guanidinium derivative of calix[4]arene was found to be more active than the corresponding monomer. On the same note, da Silva et al. (2016) also reported that iminecalix[4]arenes showed better inhibition potential against *Candida* strains than their corresponding monomers.

Azo calix[4]arene having a 2-methyl-4-aminobenzoic acid unit (**COX**) was expected to show better activity than others as it is showing close resemblance with *p*ABA. However, there was no remarkable difference in the MIC value compared to compounds **SCM** and **SGC**. Minimum bactericidal concentration (MBC) was evaluated for *B. subtilis* and found to be 62.5 μg/mL. Unlike **SCM** and **SGC**, **COX** did not show bactericidal activity for the rest of the strains at 62.5 μg/mL. The antimicrobial agent is considered to be bacteriostatic when the MBC/MIC ratio is >4, while it is considered to be bactericidal if the MBC/MIC ratio is <4 (Barry et al., 1999; Pannu et al., 2011). Only one compound (**SGC)** exhibited bactericidal activity against *B. subtilis* (MBC/MIC = 2). The rest of the compounds did not show bactericidal activity against any species.

The tetrakis azo products (**ISO** and **STF**) have limited inhibitory activity. The MBC values for these two compounds were not determined because of their high MIC values. Compound **ISO** showed moderate activity against *S. epidermidis* and *E. faecalis* at MIC 62.5 μg/mL and 15.6 μg/mL, respectively. In addition, it showed similar effect like compound **COX** against *P. aeruginosa* with MIC value 62.5 μg/mL.

### *In silico* study

The synthesized azo calix[4]arenes were docked against two receptors of penicillin-binding proteins (PDB IDs: 4CJN, 1CEF) and one receptor of the viral enzyme, neuraminidase, (PDB ID: 3TI6). Penicillin-binding proteins (PBPs) are the extracellular membrane-bound enzymes found in bacteria while absent in mammalian cells (Gullo, 1994; Onoabedje et al., 2016). Inhibition of PBPs causes structural abnormalities of a bacterial cell that result in cell death and lysis and, therefore, PBPs act as validated targets for antibacterial therapy. Neuraminidase is a glycoside hydrolase enzyme required for the viral replication (Chen et al., 2016). Neuraminidase inhibitors are designed to restrain the release of the influenza virus from the infected host cells (Hariono et al., 2016).

**Figure 3:**
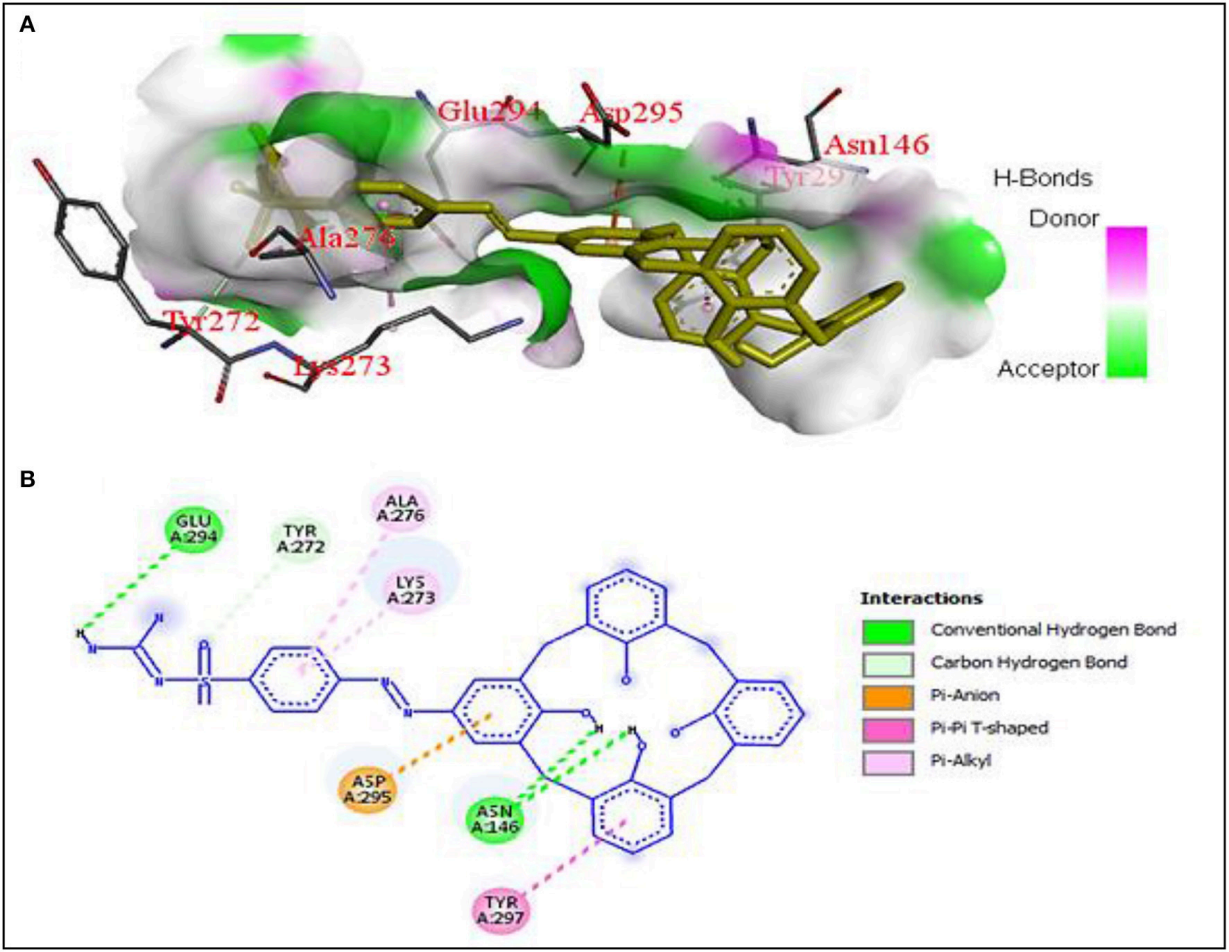
**(A)** Hydrogen bonding interactions between 4CJN and **SGC** residues with binding affinity of −9.54 kcal/mol, **(B)** 2D diagram of **SGC** interaction with 4CJN.

The values of binding energy (B.E.) and an inhibition constant of the synthesized compounds are given in Table 2. The co-crystalized ligands (**Co-lig**), 4CJN and 1CEF were re-docked in the active sites of their respective receptors and the results were taken as reference for the subsequent docking of the synthesized compounds. In the case of 3TI6, the binding affinity of azo calix[4]arenes was compared with a standard drug, oseltamivir (**OS**). Compounds containing sulfonamide group (**SGC** and **SCM**) showed more affinity toward 4CJN. The strongest interaction was shown by sulfaguanidine-based azo calix[4]arene (**SGC**) with a binding affinity of 9.54 kcal/mol and Ki value 0.10 μM which is 2.8 times stronger than the co-crystallized ligand. In general, drugs that show Ki value <1 mM are considered to be effective agents(Karimi, 2014). Hydrogen bond interaction was observed between the phenolic hydroxyl group of calix[4]arene unit and Asn146. A second hydrogen bond is formed by guanidinium part with Glu294. Lys273, which has been reported as the key residue in the active site of 4CJN, showed hydrophobic interaction with the aromatic ring of sulfaguanidine moiety (de Araújo et al., 2016) (Figure 3). **SCM** showed comparable result with the co-crystallized ligand with binding energy −8.97 kcal/mol and Ki value 0.26 μM, followed by **COX** (−8.68 kcal/mol), **ISO** (−8.54 kcal/mol) and **STF** (−8.00 kcal/mol).

**Table 2:** List of binding energy (B.E.) and inhibition constant (Ki) of synthesized compounds and co-crystalized ligand(Co-lig)/OS as calculated using molecular docking.

**Ligands**	**4CJN**		**1CEF**		**3TI6**	
	**B.E. (kcal/mol)**	**Ki (μM)**	**B.E. (kcal/mol)**	**Ki (μM)**	**B.E. (kcal/mol)**	**Ki (μM)**
**SGC**	−9.54	0.10	−8.18	1.00	−7.07	6.59
**SCM**	−8.97	0.26	−7.69	2.32	−7.64	2.50
**COX**	−8.68	0.43	−6.59	14.75	−7.72	2.21
**SPh**	−6.75	11.33	−6.52	16.55	−5.3	52
**ISO**	−8.54	0.54	−6.67	12.83	−5.70	66.68
**STF**	−8.00	1.36	−4.44	559.93	−6.41	19.92
**Co-lig**	−8.94	0.28	−7.06	6.72	–	–
**OS**	–	–	–	–	−7.78	1.98

Compounds containing sulphonamide group (**SGC** and **SCM**) also showed better interaction with 1CEF. The binding energy for **SGC** was found to be −8.18 kcal/mol (Ki = 1.00 μM) followed by **SCM** with binding energy −7.69 kcal/mol (Ki = 2.32 μM). The corresponding co-crystallized ligand has B.E. −7.06 kcal/mol and Ki 6.72 μM. In contrast with 4CJN, **COX** has low affinity and gave higher binding energy with 1CEF. The respective azo monomer of sulfaguanidine (**SPh**) has binding energy of −6.75 kcal/mol (Ki = 11.33 μM) and −6.52 kcal/mol (Ki = 16.55 μM) with 4CJN and 1CEF, respectively. The results are in accordance with *in vitro* study where **SGC** showed higher antimicrobial activity than **SPh**.

**Figure 4:**
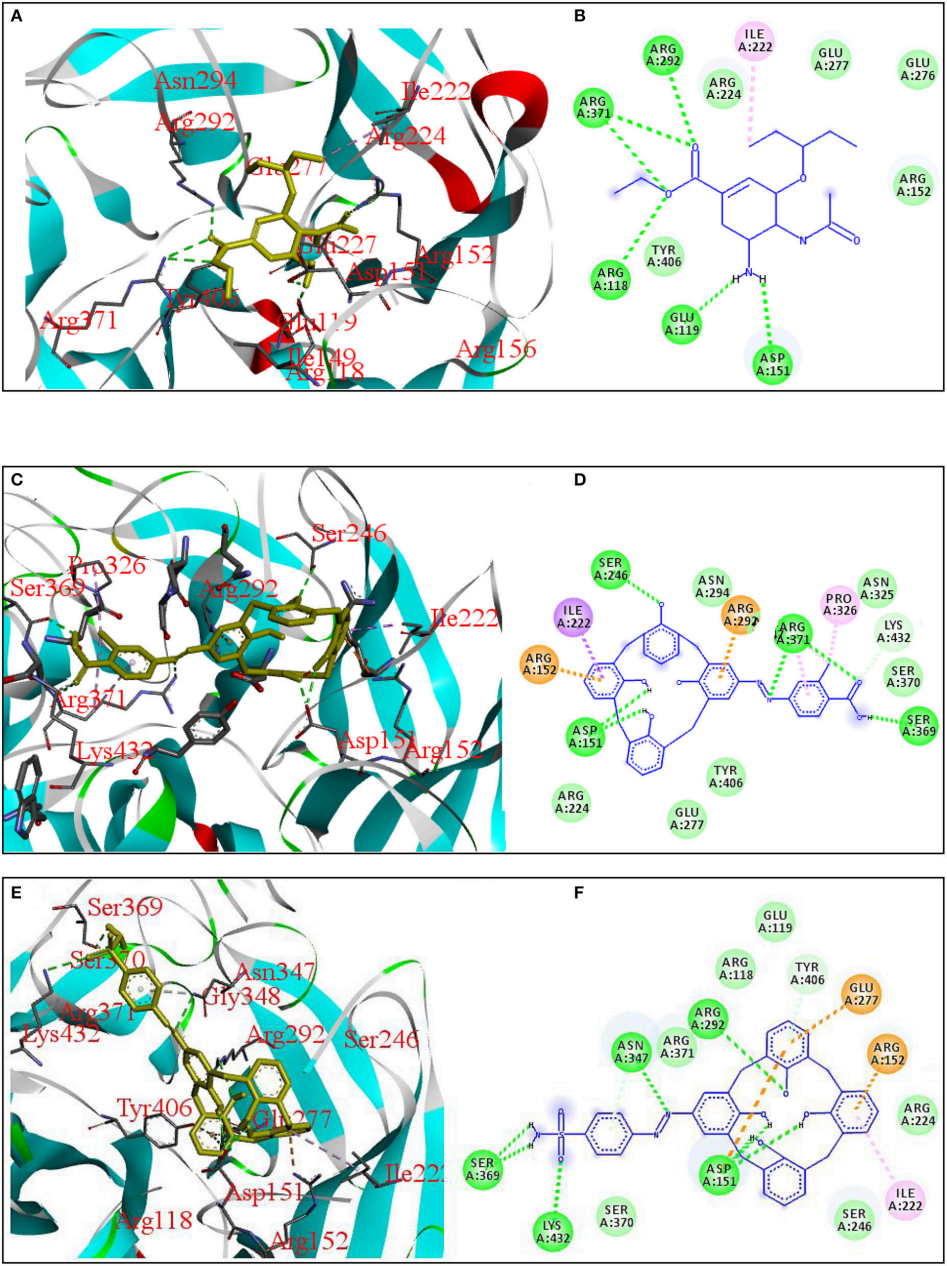
3D and 2D diagrams showing interactions of neuraminidase receptor (PDB ID: 3TI6) with **OS (A,B)**, **COX (C,D)**, and **SCM (E,F)**, respectively.

Antiviral activity of these compounds was evaluated by docking the synthesized azo calix[4]arenes against neuraminidase receptor (PDB ID: 3TI6) and four compounds showed comparable results with oseltamivir (**OS**), a standard drug used as neuraminidase inhibitor. The interactions of the two most active compounds compared to **OS** are given in Figure 4. Compound **COX** showed the highest affinity with B.E −7.72 kcal/mol that is close to the **OS** (B.E = −7.78 kcal/mol). The compound fitted well into the binding pocket, forming hydrophobic as well as hydrogen bonding interactions with Arg150, Ile22, Arg292, Ser246, Ser369, and Asp151. Sulfanilamide-based azo calix[4]arene (**SCM**) also showed good interaction with 3TI6 with a binding affinity of −7.64 kcal/mol and Ki value of 2.50 μM, followed by **SGC** with a binding affinity of −7.07 kcal/mol. The amino acid residues involved in the interactions with **SCM** include Arg292, Asp 151, and Ile222.

## Conclusion

Appropriate experimental conditions facilitated the synthesis of mono-substituted azo calix[4]arenes in good yield. The antimicrobial assay revealed that the compounds obtained from sulphanilamide, sulfaguanidine and 2-methyl-4-aminobenzoic acid showed bacteriostatic and/or bactericidal activity against *B. subtilis*, MRSA *S. aureus, S. epidermidis* and *E. faecalis* with MIC values ranging from 0.97 to 62.5 μg/mL. It suggests that azo derivatives of calix[4]arene synthesized from therapeutic agents have better antimicrobial activity compared to the parent compound. Molecular docking study against the selected microbial enzymes showed good interaction with the active site residues through hydrogen, hydrophobic and pi-pi interactions and could inhibit the activity of these enzymes. The high binding affinity of sulfaguanidine and sulphanilamide-based azo calix[4]arenes toward the targeted receptors correlates with antimicrobial assay results. The positive aspect of these results is the activity of some azo calix[4]arenes against the multidrug-resistant *Staphylococcus aureus*. However, the compounds showed low to no activity against Gram-negative and fungal strains. The area is still new and more basic and mechanistic studies are needed to explore and extend the biomedical applications of azo calixarenes, from therapeutic agents to drug carriers. Suitable functionalization and insertion of therapeutic moieties on the basic scaffold of calixarene may further enhance their bioactivity.

## Author contributions

SA: supervised the research project; YA: synthesized the compounds and performed modeling and *in vitro* study; AM: assisted in molecular docking; NM and DS: provided technical support.

### Conflict of interest statement

The authors declare that the research was conducted in the absence of any commercial or financial relationships that could be construed as a potential conflict of interest.
