# Supplementary material for: Synthesis, in-Vitro and in Silico Studies of Azo-Based Calix[4]arenes as Antibacterial Agent and Neuraminidase Inhibitor: A New Look Into an Old Scaffold
Source: Front Chem. 2018 Jun 12;6:210. doi: 10.3389/fchem.2018.00210 (PMC6005842; doi:10.3389/fchem.2018.00210)
Supplement: Supplementary file 1 [file Data_Sheet_1.PDF]

SUPPORTING INFORMATION

SYNTHESIS, IN-VITRO AND *IN SILICO* STUDIES OF AZO-BASED CALIX[4]ARENES AS ANTIBACTERIAL AGENT AND NEURAMINIDASE INHIBITOR: A NEW LOOK INTO AN OLD SCAFFOLD

Yousaf Ali[1,2], Noraslinda Mohamad Bunnori[2], Deny Susanti[2], Alhassan Muhammad Alhassan[3] & Shafida Abd Hamid[2]*

[1]Department of Chemistry, Sarhad University of Science and Information Technology, Peshawar, Pakistan
[2]Kulliyyah of Science, International Islamic University Malaysia, Bandar Indera Mahkota, 25200 Kuantan, Malaysia
[3]Kulliyah of Pharmacy, International Islamic University Malaysia, Bandar Indera Mahkota, 25200 Kuantan, Malaysia

***Correspondance**:

Dr Shafida Abd Hamid
E-mail: shafida@iium.edu.my
Tel: +60 9570 5003; Fax: +60 9571 571 6783

1. 25,26,27,28-Tetrahydroxyclaix[4]arene

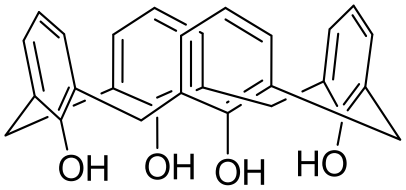

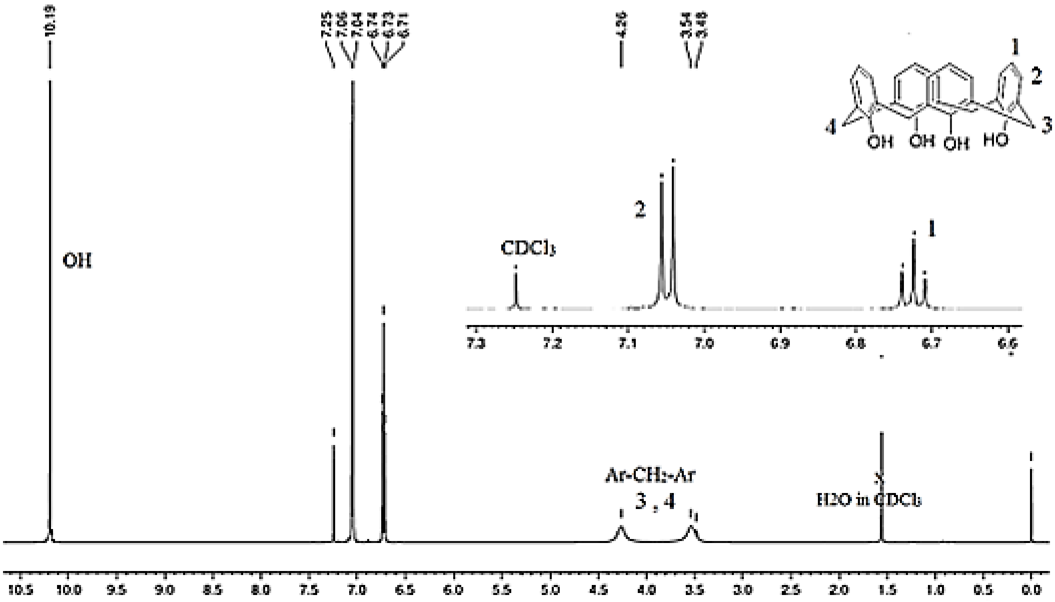

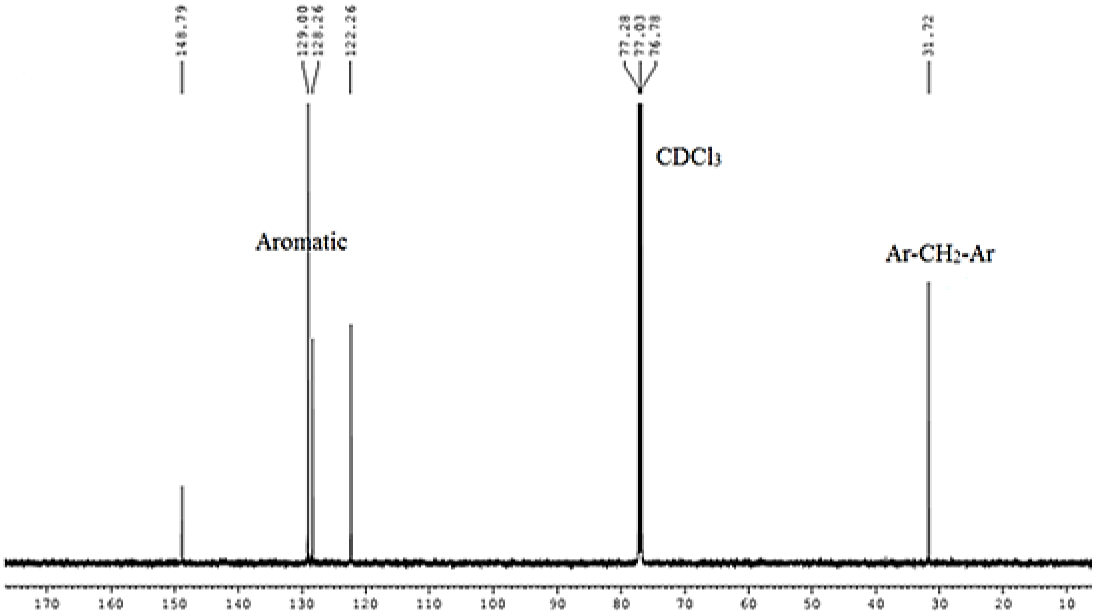

2. **Synthesis of *N*-(diaminomethylidene)-4-[(*E*)-(4- hydroxyphenyl)diazenyl] benzenesulfonamide (SPh)**

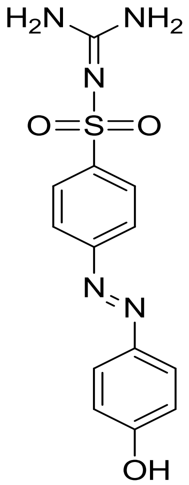

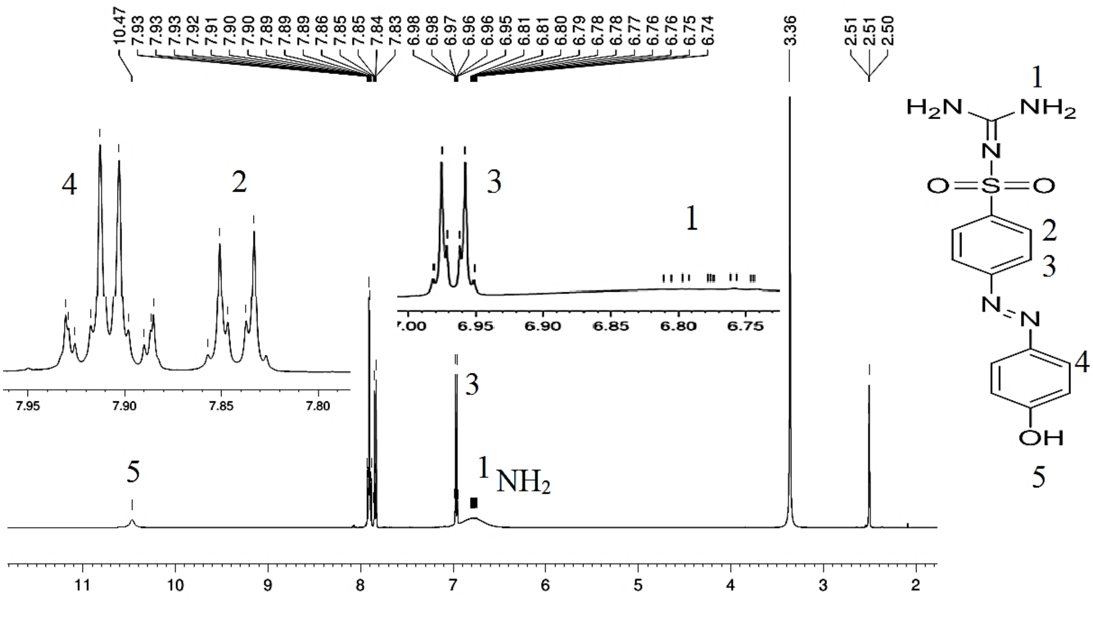

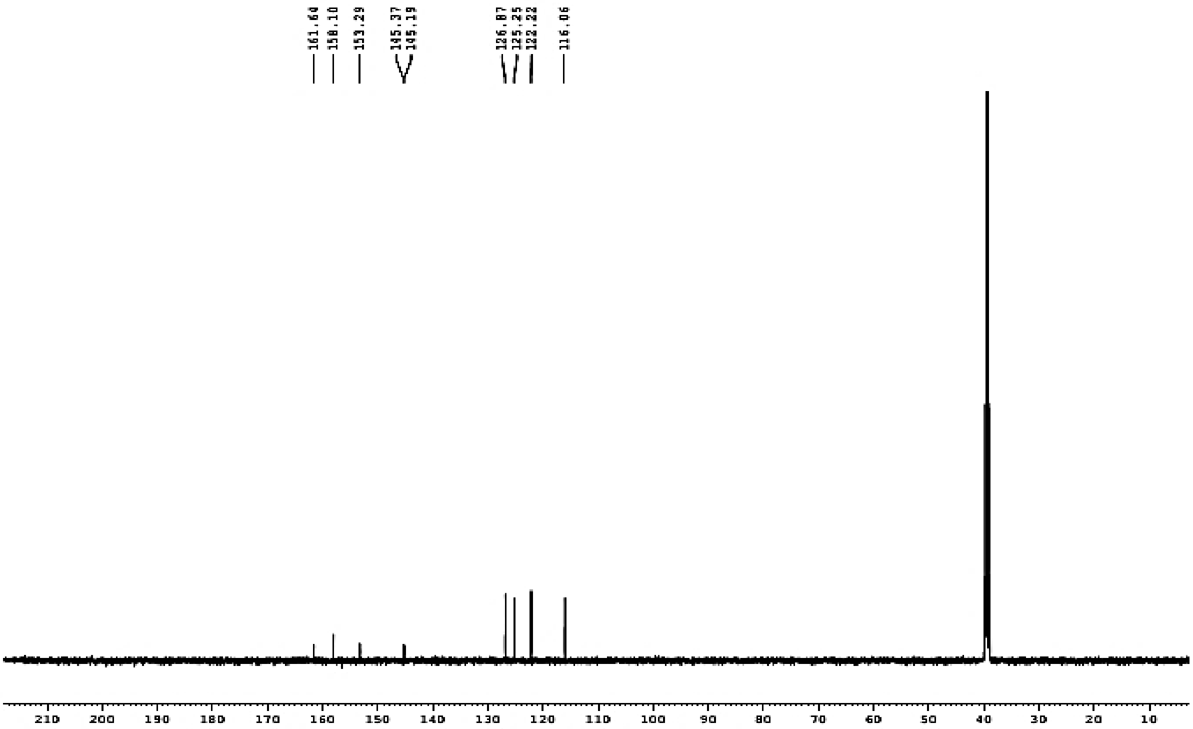

3. 25,26,27,28-Tetrahydroxy-5-(sulfaguanidine)azocalix[4]arene (SGC)

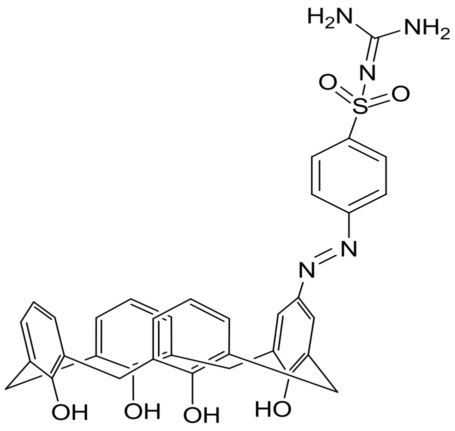

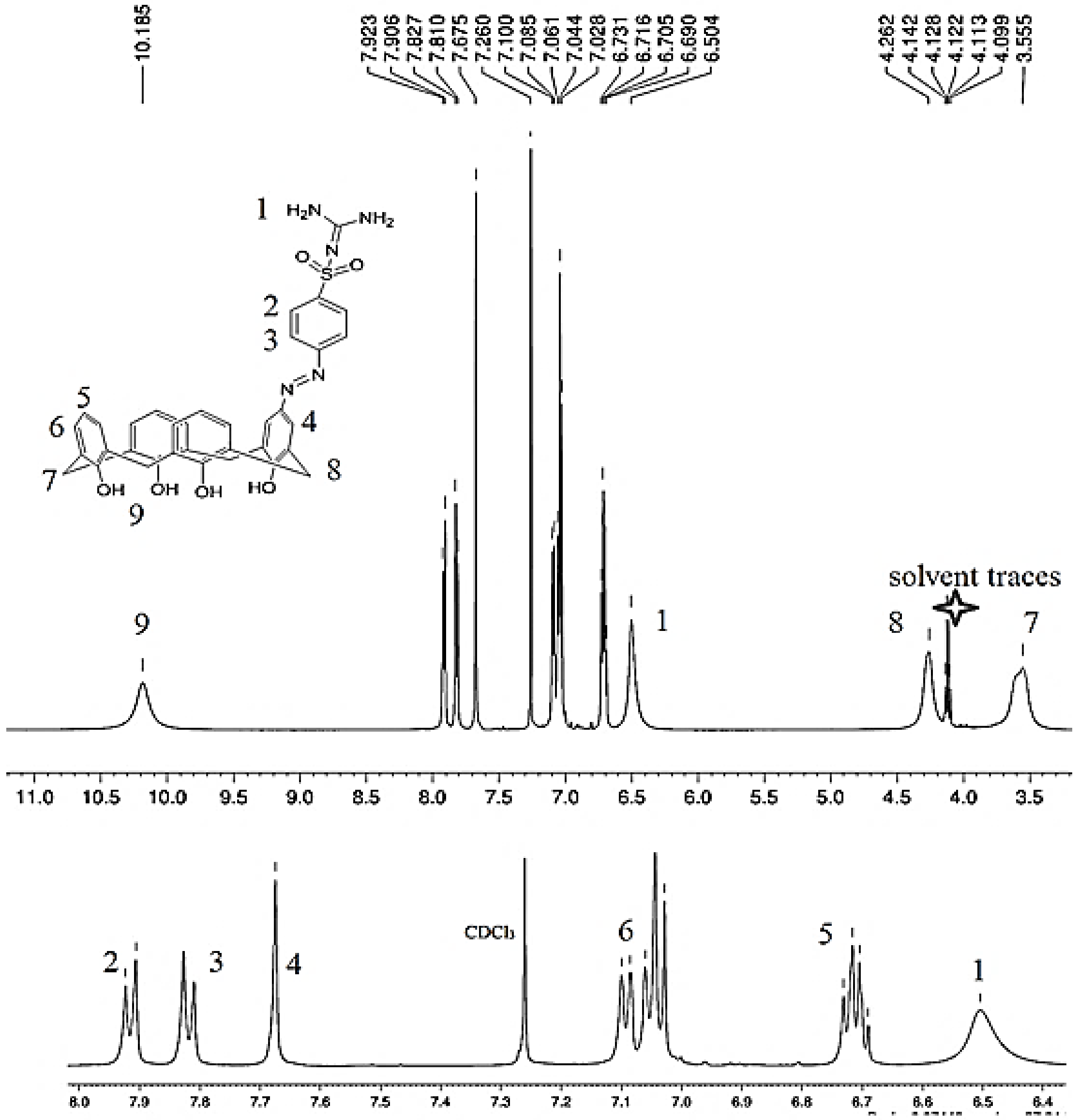
10.185
7.923
7.906
7.827
7.810
7.675
7.260
7.100
7.085
7.061
7.044
7.028
6.731
6.716
6.705
6.690
6.504
4.262
4.142
4.128
4.122
4.113
4.099
3.555
H2N
NH2
N
O
S
O
N
N
OH
OH
OH
HO
1
2
3
4
5
6
7
8
9
solvent traces
CDCl3

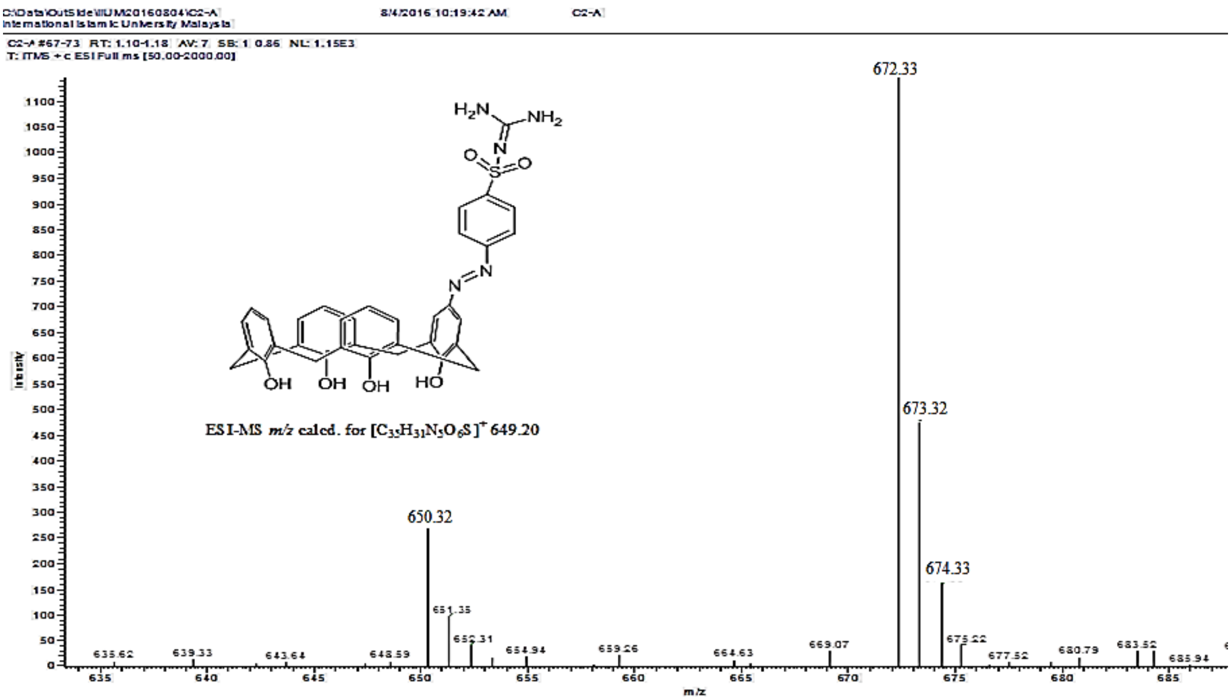

4. 25,26,27,28-Tetrahydroxy-5-(4-sulphonylaminophenyl)azocalix[4]arene (CM)

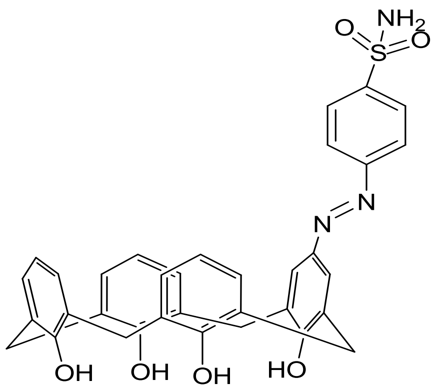

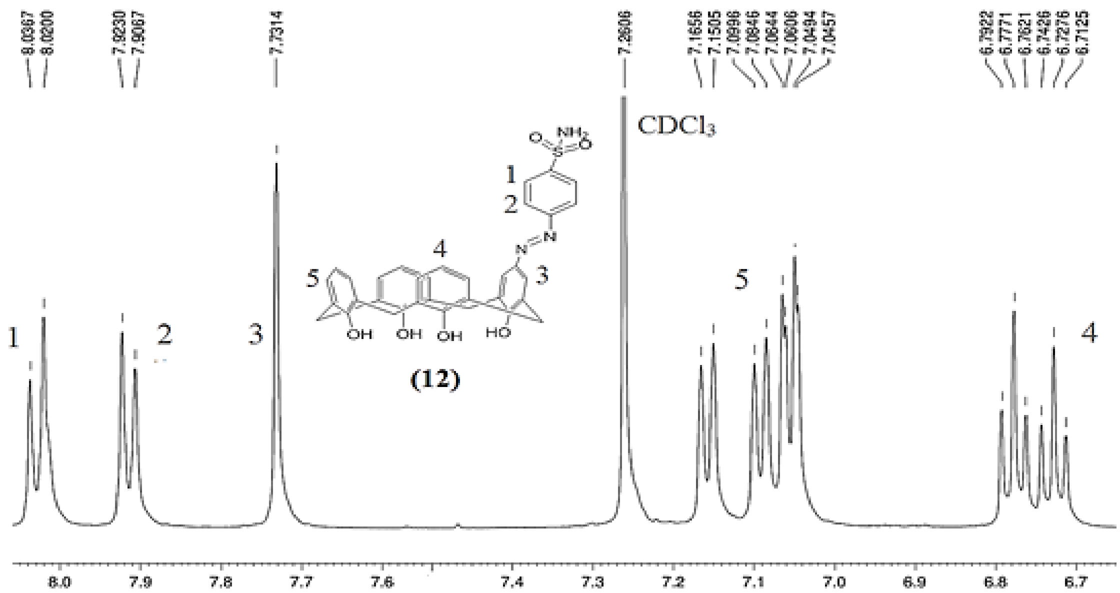
8.0367
8.0200
7.9230
7.9067
7.7314
7.2606
7.1656
7.1505
7.0996
7.0846
7.0644
7.0606
7.0494
7.0457
6.7922
6.7771
6.7621
6.7426
6.7276
6.7125
CDCl3
NH2
O
S
O
N
N
OH
OH
OH
HO
1
2
3
4
5
(12)
8.0
7.9
7.8
7.7
7.6
7.4
7.3
7.2
7.1
7.0
6.9
6.8
6.7

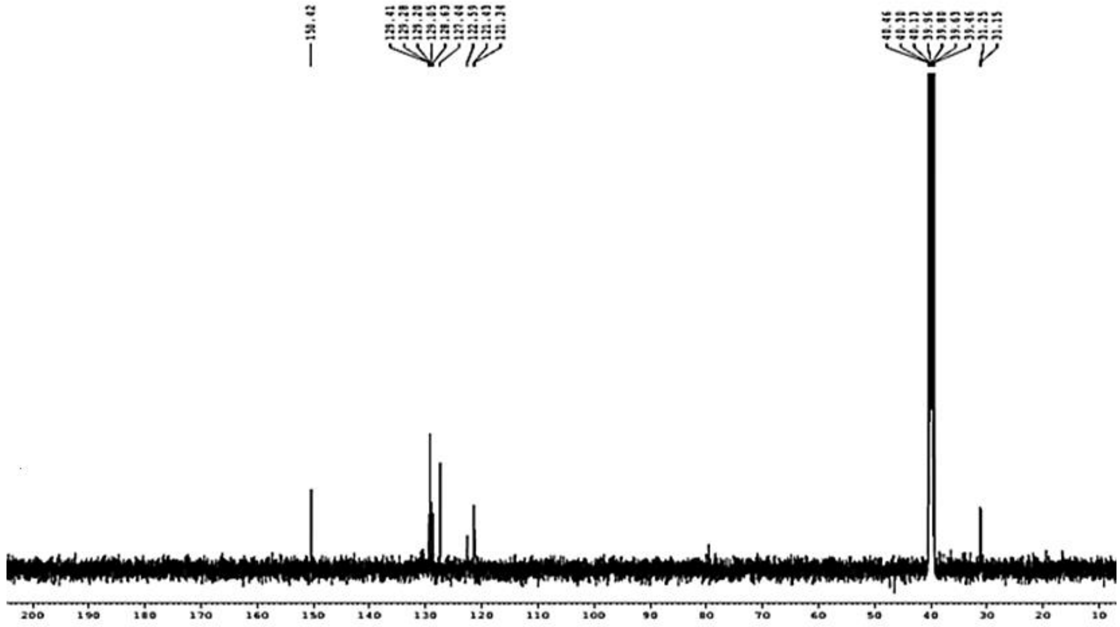
200
190
180
170
160
150
140
130
120
110
100
90
80
70
60
50
40
30
20
10

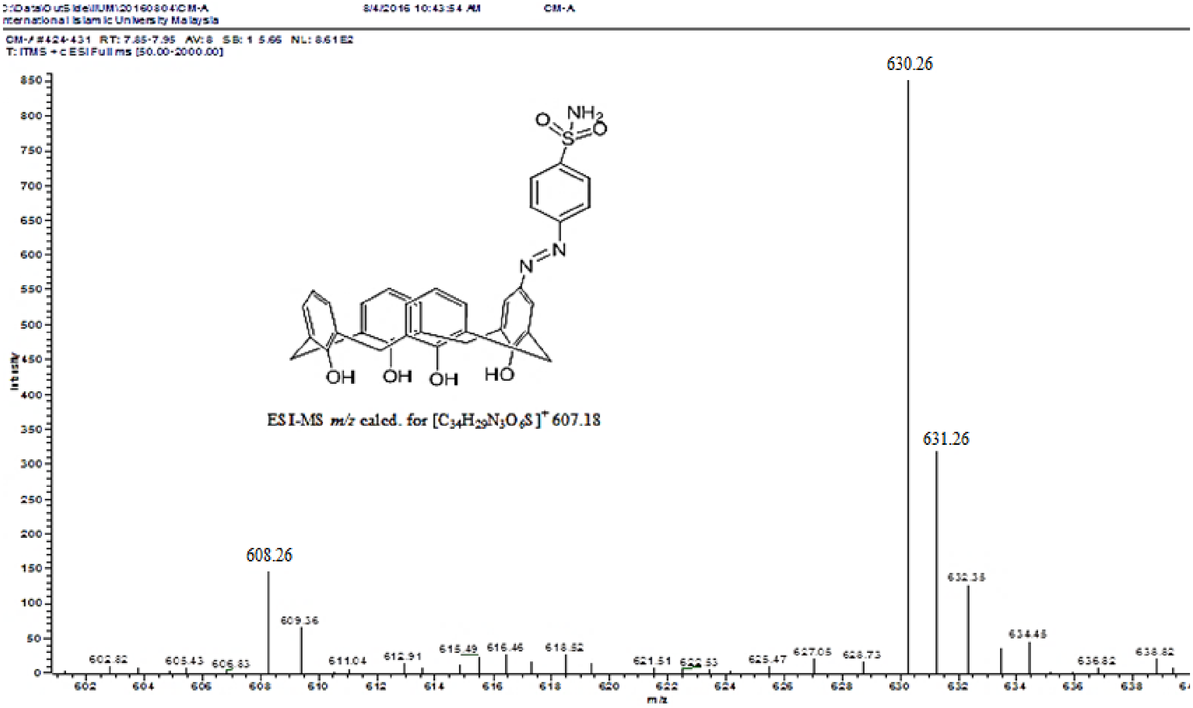

5. **25,26,27,28-Tetrahydroxy-5-((4-corboxy-3-methyl)phenyl)azocalix[4]arene (COX)**

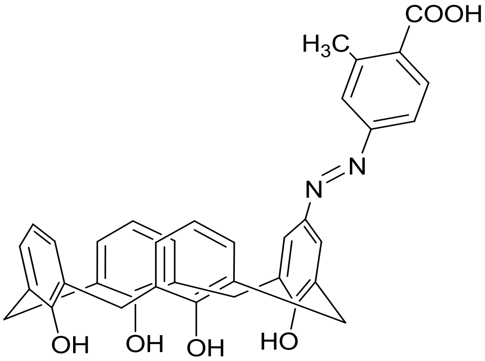

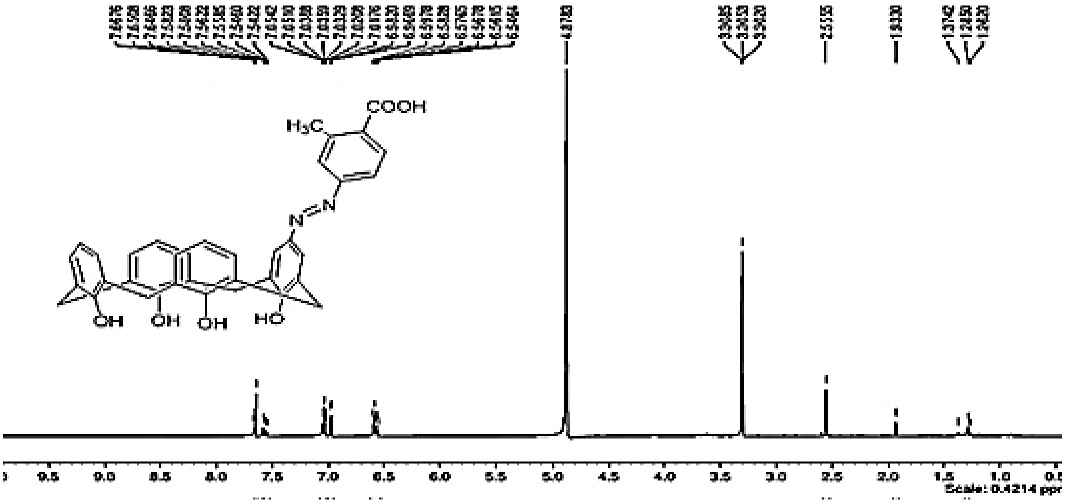
COOH
OH
OH
OH
HO

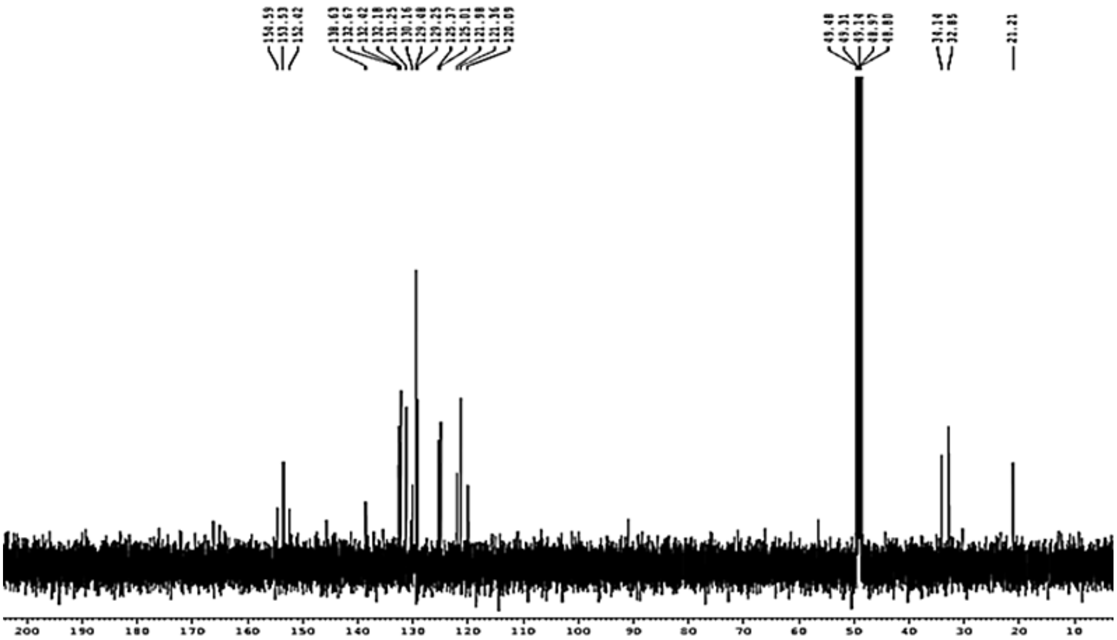

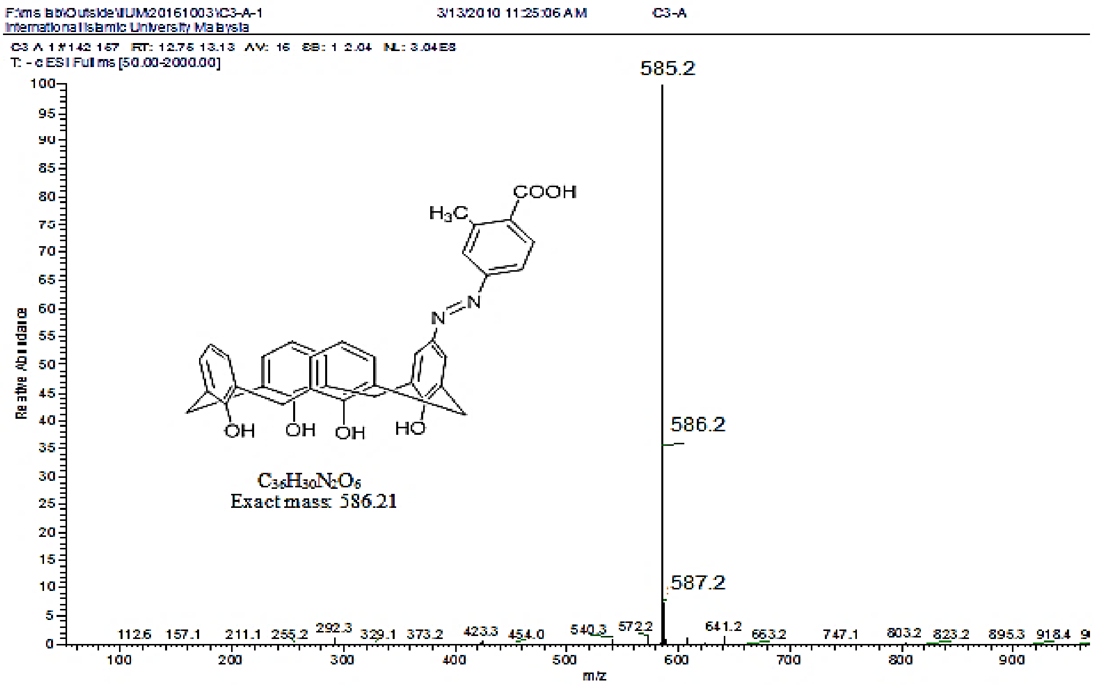

6. 25,26,27,28-Tetrahydroxy-5,11,17,23-tetrakis(3-trifluoromethyl)phenyl) azocalix[4]arene (STF)

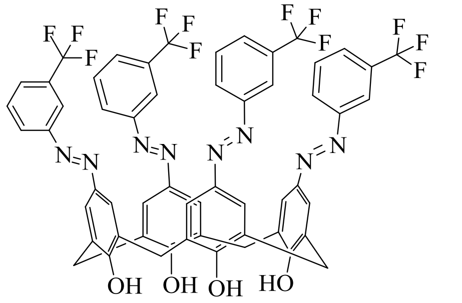

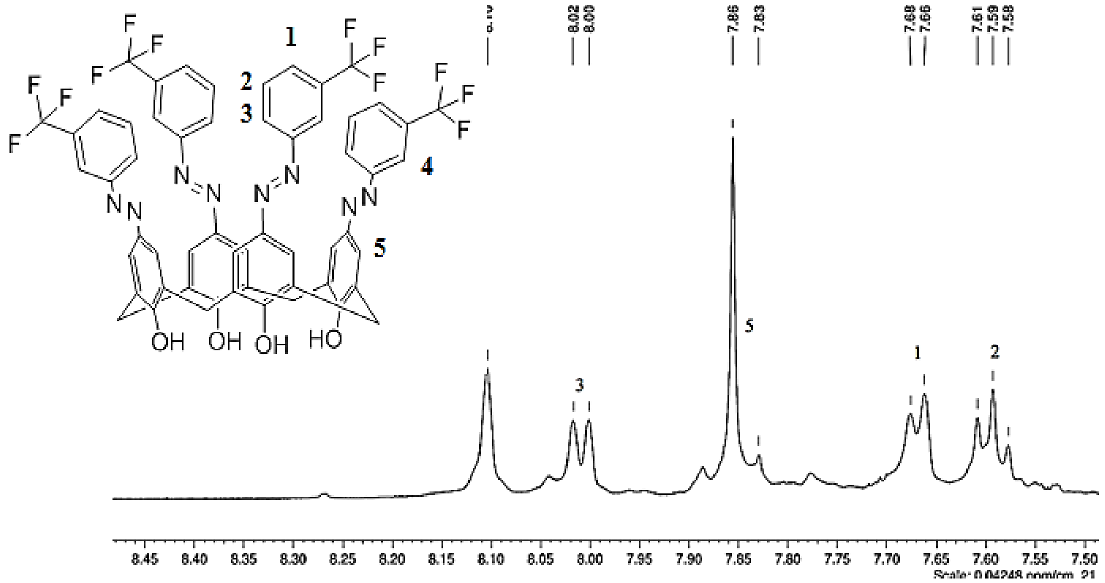
8.02
8.00
7.86
7.83
7.68
7.66
7.61
7.59
7.58
1
2
3
4
5
OH
HO
8.45 8.40 8.35 8.30 8.25 8.20 8.15 8.10 8.05 8.00 7.95 7.90 7.85 7.80 7.75 7.70 7.65 7.60 7.55 7.50

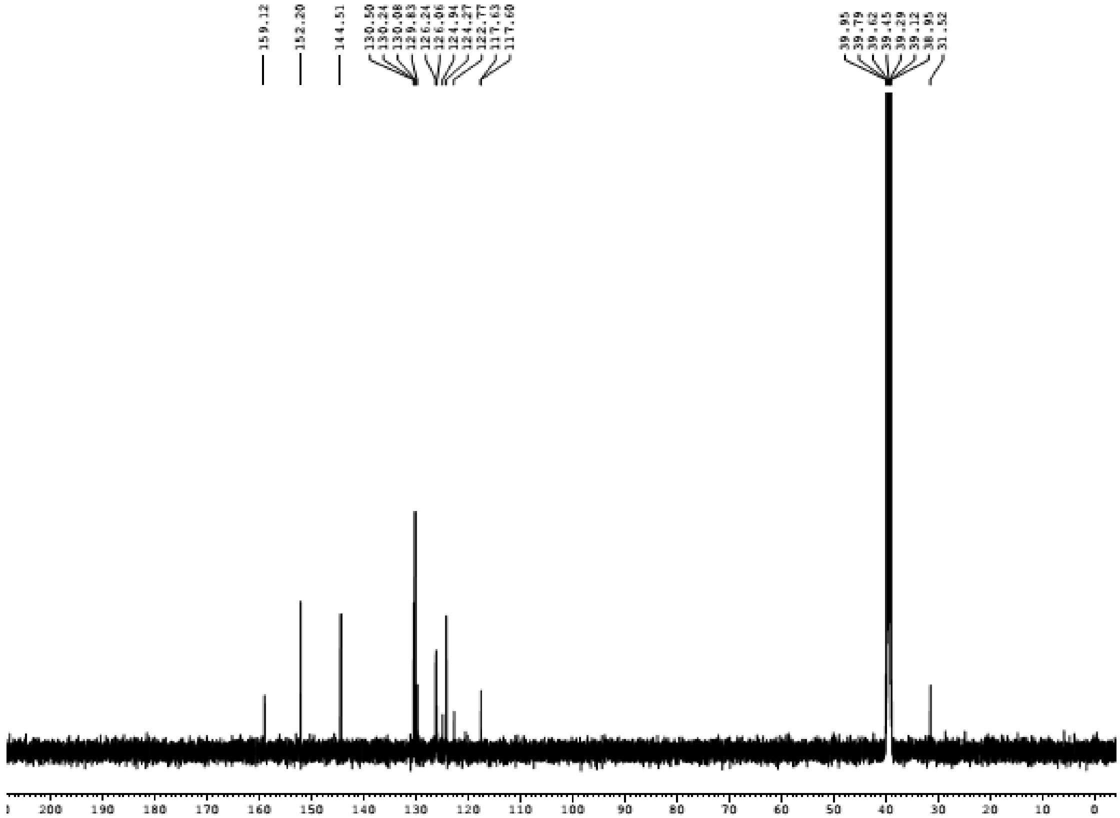
159.12
152.20
144.51
130.50
130.24
130.08
129.83
126.24
126.06
124.94
124.27
122.77
117.63
117.60
39.95
39.79
39.62
39.45
39.29
39.12
38.95
31.52
200 190 180 170 160 150 140 130 120 110 100 90 80 70 60 50 40 30 20 10 0

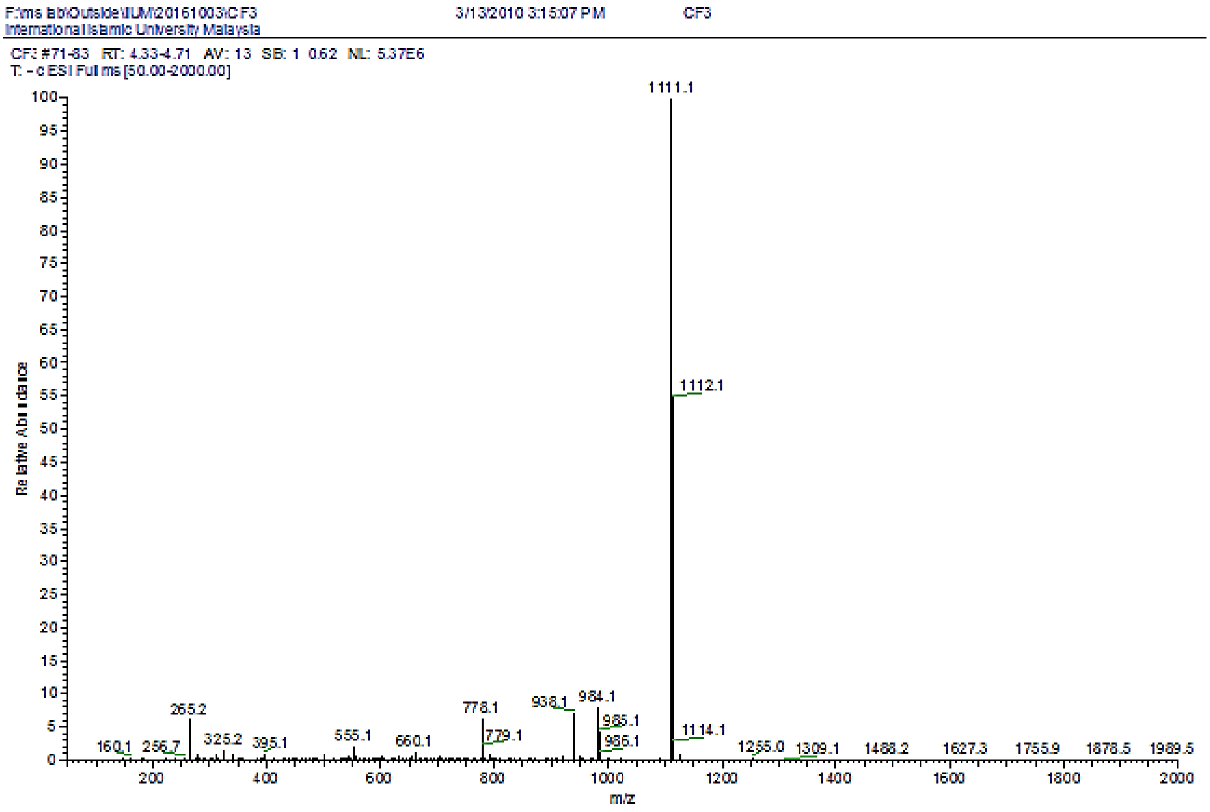

7. 25,26,27,28-Tetrahydroxy-5,11,17,23-tetra(4-isopropylphenyl)azo calix[4]arene (ISO)

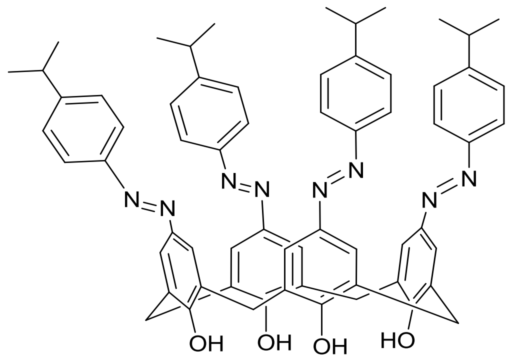

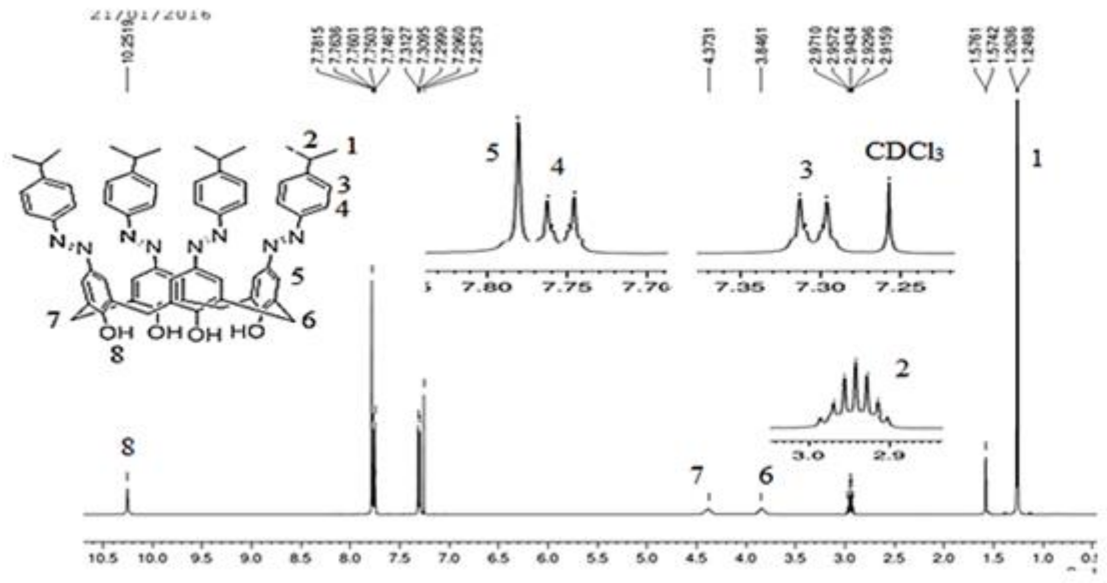
CDCl3
OH OH OH HO
Aromatic
6/7

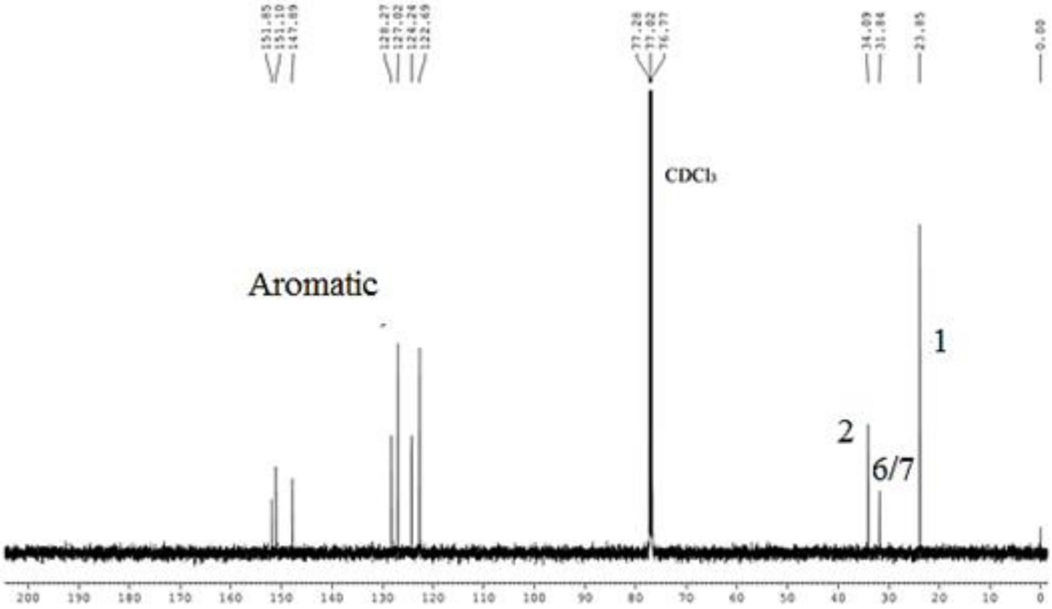
CDCl3
Aromatic
6/7

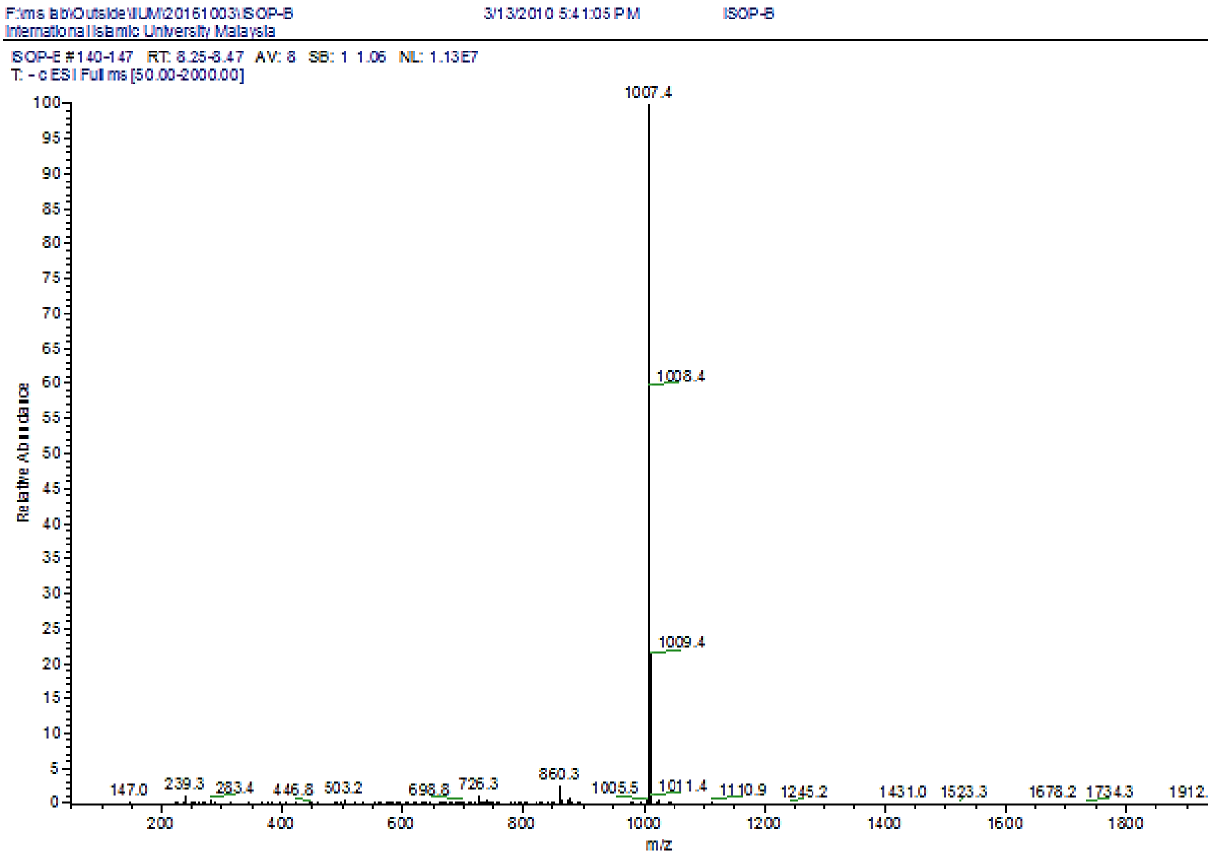
F:\ms lab\Outside\IIUM\20161003\ISOP-B
3/13/2010 5:41:05 PM
ISOP-B
International Islamic University Malaysia
ISOP-B #140-147 RT: 8.25-8.47 AV: 8 SB: 1 1.06 NL: 1.13E7
T: - c ESI Full ms [50.00-2000.00]
Relative Abundance
m/z
1007.4
1008.4
1009.4
147.0
239.3
283.4
446.8
503.2
698.8
726.3
860.3
1005.5
1011.4
1110.9
1245.2
1431.0
1523.3
1678.2
1734.3
1912.
